# Bis(2-amino-3*H*-benzothia­zolium) bis­(7-oxabicyclo­[2.2.1]heptane-2,3-di­carbox­yl­ato)manganate(II) hexa­hydrate

**DOI:** 10.1107/S160053681002091X

**Published:** 2010-06-09

**Authors:** Na Wang, Yi-Hang Wen, Qiu-Yue Lin, Jie Feng

**Affiliations:** a Zhejiang Key Laboratory for Reactive Chemistry on Solid Surfaces, Institute of Physical Chemistry, Zhejiang Normal University, Jinhua, Zhejiang 321004, People’s Republic of China; b College of Chemistry and Life Science, Zhejiang Normal University, Jinhua 321004, Zhejiang, People’s Republic of China

## Abstract

In the crystal structure of the title salt, (C_7_H_7_N_2_S)_2_[Mn(C_8_H_8_O_5_)_2_]·6H_2_O, the heterocyclic N atom of the 2-amino­benzothia­zole mol­ecule is protonated. The Mn^II^ atom (site symmetry 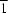) has a slightly distorted octa­hedral MnO_6_ coordination defined by the bridging O atoms of the bicyclo­heptane unit and four carboxyl­ate O atoms of two symmetry-related and fully deprotonated ligands. The crystal packing is stabilized by N—H⋯O hydrogen bonds between the cations and anions and by O—H⋯O hydrogen bonds including the crystal water mol­ecules.

## Related literature

7-Oxabicyclo­[2.2.1]heptane-2,3-dicarb­oxy­lic anhydride (nor­cantharidin) is a lower toxicity anti­cancer drug, see: Shimi *et al.* (1982 ▶). Manganese is a cofactor or required metal ion for many enzymes, see: Dukhande *et al.* (2006 ▶). For the isotypic structure of the Co analogue, see: Wang *et al.* (2010 ▶).
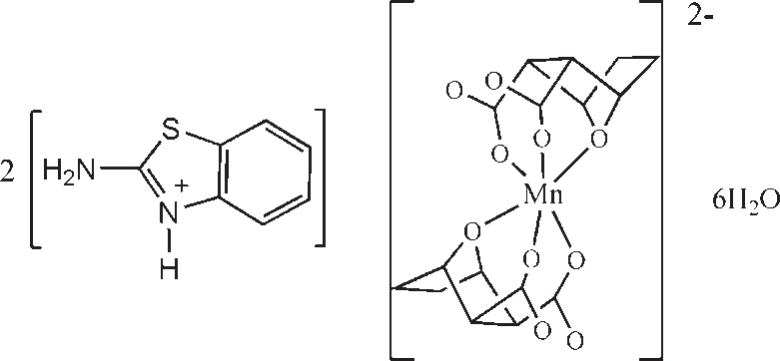


## Experimental

### 

#### Crystal data

(C_7_H_7_N_2_S)_2_[Mn(C_8_H_8_O_5_)_2_]·6H_2_O
*M* *_r_* = 833.76
Triclinic, 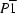

*a* = 6.6937 (1) Å
*b* = 10.2209 (1) Å
*c* = 13.1163 (2) Å
α = 89.527 (1)°
β = 88.831 (1)°
γ = 81.514 (1)°
*V* = 887.34 (2) Å^3^
*Z* = 1
Mo *K*α radiation
μ = 0.57 mm^−1^
*T* = 296 K
0.15 × 0.13 × 0.10 mm

#### Data collection

Bruker APEXII area-detector diffractometer
Absorption correction: multi-scan (*SADABS*; Sheldrick, 1996 ▶) *T*_min_ = 0.916, *T*_max_ = 0.944
14148 measured reflections
4089 independent reflections
3426 reflections with *I* > 2σ(*I*)
*R*_int_ = 0.026

#### Refinement

*R*[*F* ^2^ > 2σ(*F* ^2^)] = 0.032
*wR*(*F* ^2^) = 0.093
*S* = 0.97
4089 reflections
262 parameters
10 restraints
H atoms treated by a mixture of independent and constrained refinement
Δρ_max_ = 0.31 e Å^−3^
Δρ_min_ = −0.35 e Å^−3^

### 

Data collection: *APEX2* (Bruker, 2006 ▶); cell refinement: *SAINT* (Bruker, 2006 ▶); data reduction: *SAINT*; program(s) used to solve structure: *SHELXS97* (Sheldrick, 2008 ▶); program(s) used to refine structure: *SHELXL97* (Sheldrick, 2008 ▶); molecular graphics: *SHELXTL* (Sheldrick, 2008 ▶); software used to prepare material for publication: *SHELXL97*.

## Supplementary Material

Crystal structure: contains datablocks I, global. DOI: 10.1107/S160053681002091X/wm2350sup1.cif

Structure factors: contains datablocks I. DOI: 10.1107/S160053681002091X/wm2350Isup2.hkl

Additional supplementary materials:  crystallographic information; 3D view; checkCIF report

## Figures and Tables

**Table 1:** Selected bond lengths (Å)

Mn1—O4	2.1083 (11)
Mn1—O2	2.1883 (11)
Mn1—O5	2.2598 (11)

**Table 2:** Hydrogen-bond geometry (Å, °)

*D*—H⋯*A*	*D*—H	H⋯*A*	*D*⋯*A*	*D*—H⋯*A*
N1—H1*N*⋯O1^i^	0.84 (2)	1.84 (2)	2.6822 (18)	178 (2)
N2—H2*B*⋯O2^i^	0.86	2.00	2.8490 (19)	170
N2—H2*C*⋯O2*W*^ii^	0.86	2.00	2.824 (2)	160
O1*W*—H1*WA*⋯O1	0.81 (2)	2.03 (2)	2.8250 (19)	166 (3)
O1*W*—H1*WB*⋯O2*W*	0.85 (2)	1.95 (2)	2.792 (2)	170 (3)
O2*W*—H2*WA*⋯O3	0.83 (2)	1.87 (2)	2.6806 (19)	169 (3)
O2*W*—H2*WB*⋯O3*W*^iii^	0.84 (2)	1.93 (2)	2.768 (2)	178 (3)
O3*W*—H3*WA*⋯O1*W*^ii^	0.80 (2)	2.21 (2)	3.004 (2)	169 (3)
O3*W*—H3*WB*⋯O1*W*	0.82 (2)	1.97 (2)	2.784 (2)	173 (3)

Symmetry codes: (i) 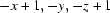; (ii) 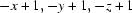; (iii) 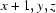.

**Fig. 1.**
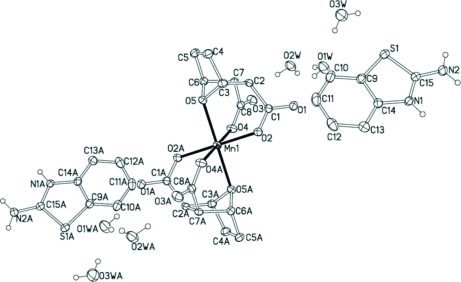
A view of the molecular units of the title salt showing the atom-labelling scheme with displacement ellipsoids drawn at the 30% probability. Symmetry code: A (-x+2, -y, -z).

## supplementary crystallographic information

### Comment

7-Oxabicyclo[2,2,1] heptane-2,3-dicarboxylic anhydride (norcantharidin) derived from cantharidin is a lower toxicity anticancer drug (Shimi *et al.*, 1982). Manganese is an important trace element needed for normal physiological functions and development. It is also a cofactor or required metal ion for many enzymes, such as superoxide dismutase, glutamine synthetase and arginase (Dukhande *et al.*, 2006).

In the title complex, (C_7_H_7_N_2_S)^+^_2_[Mn(C_8_H_8_O_5_)_2_]^2-^(H_2_O)_6_, the Mn^II^ ion is located on a crystallographic centre of inversion. Two bridging oxygen atoms of the bicycloheptane units and four carboxylate oxygen atoms give rise to a slightly distorted octahedral coordination environment around the Mn^II^ atom. The bond angles O2—Mn1—O2^i^, O4—Mn1—O4^i^ and O5—Mn1—O5^i^ (i: -x+2, -y, -z) are 180°, while the bond angles O4—Mn1—O2 and O2—Mn1—O4^i^ open up slightly from 86.35 (5)° to 93.65 (5)°, resulting in a slight distortion from the ideal octahedral geometry. The crystal packing is stabilized by N—H···O hydrogen bonds between the cations and anions and by O—H···O hydrogen bonds including the crystal water molecules.

The crystal structure of (C_7_H_7_N_2_S)^+^_2_[Mn(C_8_H_8_O_5_)_2_]^2-^(H_2_O)_6_ is isotypic with that of the Co analogue (Wang *et al.*, 2010) where slightly shorter metal—oxygen bonds are observed.

### Experimental

Norcantharidin, manganese acetate and 2-aminobenzothiazole were dissolved in 15 mL distilled water. The mixture was sealed in a 25 mL Teflon-lined stainless vessel and heated at 443 K for 3 d, then cooled slowly to room temperature. Colourless crystals suitable for X-ray diffraction were obtained.

### Refinement

The H atoms bonded to C and N atoms were positioned geometrically and refined using a riding model [aromatic C—H = 0.93 Å, aliphatic C—H = 0.97–0.98 Å and N—H = 0.86 Å and *U*_iso_(H)=1.2U_eq_(parent atom)]. The H atoms of the water molecule were located in a difference Fourier maps and refined with O—H distance restraints of 0.85 (2) and *U*_iso_(H) = 1.5U_eq_(O).

### Crystal data

**Table:**

(C_7_H_7_N_2_S)_2_[Mn(C_8_H_8_O_5_)_2_]·6H_2_O	*Z* = 1
*M**_r_* = 833.76	*F*(000) = 435
Triclinic, *P*1	*D*_x_ = 1.560 Mg m^−^^3^
Hall symbol: -P 1	Mo *K*α radiation, λ = 0.71073 Å
*a* = 6.6937 (1) Å	Cell parameters from 5204 reflections
*b* = 10.2209 (1) Å	θ = 1.6–27.6°
*c* = 13.1163 (2) Å	µ = 0.57 mm^−^^1^
α = 89.527 (1)°	*T* = 296 K
β = 88.831 (1)°	Block, colourless
γ = 81.514 (1)°	0.15 × 0.13 × 0.10 mm
*V* = 887.34 (2) Å^3^	

### Data collection

**Table:**

Bruker APEXII area-detector diffractometer	4089 independent reflections
Radiation source: fine-focus sealed tube	3426 reflections with *I* > 2σ(*I*)
graphite	*R*_int_ = 0.026
ω scans	θ_max_ = 27.6°, θ_min_ = 1.6°
Absorption correction: multi-scan (*SADABS*; Sheldrick, 1996)	*h* = −8→8
*T*_min_ = 0.916, *T*_max_ = 0.944	*k* = −12→13
14148 measured reflections	*l* = −17→17

### Refinement

**Table:**

Refinement on *F*^2^	Primary atom site location: structure-invariant direct methods
Least-squares matrix: full	Secondary atom site location: difference Fourier map
*R*[*F*^2^ > 2σ(*F*^2^)] = 0.032	Hydrogen site location: inferred from neighbouring sites
*wR*(*F*^2^) = 0.093	H atoms treated by a mixture of independent and constrained refinement
*S* = 0.97	*w* = 1/[σ^2^(*F*_o_^2^) + (0.0536*P*)^2^ + 0.2749*P*] where *P* = (*F*_o_^2^ + 2*F*_c_^2^)/3
4089 reflections	(Δ/σ)_max_ = 0.001
262 parameters	Δρ_max_ = 0.31 e Å^−^^3^
10 restraints	Δρ_min_ = −0.34 e Å^−^^3^

### Special details

**Table:**

Geometry. All e.s.d.'s (except the e.s.d. in the dihedral angle between two l.s. planes) are estimated using the full covariance matrix. The cell e.s.d.'s are taken into account individually in the estimation of e.s.d.'s in distances, angles and torsion angles; correlations between e.s.d.'s in cell parameters are only used when they are defined by crystal symmetry. An approximate (isotropic) treatment of cell e.s.d.'s is used for estimating e.s.d.'s involving l.s. planes.
Refinement. Refinement of *F*^2^ against ALL reflections. The weighted *R*-factor *wR* and goodness of fit *S* are based on *F*^2^, conventional *R*-factors *R* are based on *F*, with *F* set to zero for negative *F*^2^. The threshold expression of *F*^2^ > σ(*F*^2^) is used only for calculating *R*-factors(gt) *etc*. and is not relevant to the choice of reflections for refinement. *R*-factors based on *F*^2^ are statistically about twice as large as those based on *F*, and *R*- factors based on ALL data will be even larger.

### Fractional atomic coordinates and isotropic or equivalent isotropic displacement parameters (Å^2^)

**Table:**

	*x*	*y*	*z*	*U*_iso_*/*U*_eq_	
Mn1	1.0000	0.0000	0.0000	0.02770 (11)	
S1	0.17382 (7)	0.26390 (4)	0.52462 (3)	0.03610 (12)	
N1	0.2239 (2)	0.03022 (14)	0.60022 (10)	0.0278 (3)	
H1N	0.236 (3)	−0.0300 (18)	0.6447 (14)	0.042*	
N2	0.1568 (2)	0.19534 (15)	0.72262 (11)	0.0369 (3)	
H2B	0.1649	0.1382	0.7714	0.044*	
H2C	0.1309	0.2784	0.7360	0.044*	
O1	0.7284 (2)	0.16252 (12)	0.25851 (8)	0.0361 (3)	
O1W	0.6844 (3)	0.39782 (16)	0.37356 (12)	0.0552 (4)	
H1WA	0.679 (4)	0.335 (2)	0.336 (2)	0.083*	
H1WB	0.777 (4)	0.440 (2)	0.352 (2)	0.083*	
O2	0.7957 (2)	0.01642 (11)	0.13318 (9)	0.0357 (3)	
O2W	0.9775 (2)	0.52942 (14)	0.28069 (12)	0.0493 (4)	
H2WA	1.013 (4)	0.480 (2)	0.2319 (15)	0.074*	
H2WB	1.076 (3)	0.534 (3)	0.3180 (17)	0.074*	
O3	1.12534 (19)	0.34977 (13)	0.14033 (10)	0.0392 (3)	
O3W	0.3055 (3)	0.5480 (2)	0.40200 (13)	0.0654 (5)	
H3WA	0.292 (5)	0.564 (3)	0.4616 (14)	0.098*	
H3WB	0.416 (3)	0.505 (3)	0.389 (2)	0.098*	
O4	1.12511 (18)	0.15814 (12)	0.06293 (10)	0.0356 (3)	
O5	0.77654 (18)	0.16207 (11)	−0.06930 (8)	0.0303 (3)	
C1	0.7329 (2)	0.13207 (16)	0.16658 (12)	0.0271 (3)	
C2	0.6576 (2)	0.24024 (15)	0.09025 (11)	0.0260 (3)	
H2A	0.5468	0.3013	0.1213	0.031*	
C3	0.5877 (2)	0.18764 (16)	−0.00995 (12)	0.0298 (3)	
H3A	0.5198	0.1095	−0.0011	0.036*	
C4	0.4672 (3)	0.29791 (17)	−0.07160 (14)	0.0356 (4)	
H4A	0.3680	0.3523	−0.0291	0.043*	
H4B	0.3994	0.2628	−0.1277	0.043*	
C5	0.6338 (3)	0.37615 (17)	−0.11049 (13)	0.0345 (4)	
H5A	0.6423	0.3773	−0.1844	0.041*	
H5B	0.6116	0.4663	−0.0856	0.041*	
C6	0.8217 (3)	0.29710 (15)	−0.06493 (11)	0.0284 (3)	
H6A	0.9469	0.3094	−0.1014	0.034*	
C7	0.8286 (2)	0.32037 (15)	0.05059 (11)	0.0254 (3)	
H7A	0.7900	0.4146	0.0654	0.030*	
C8	1.0415 (2)	0.27264 (16)	0.08941 (12)	0.0279 (3)	
C9	0.2250 (3)	0.12947 (18)	0.44134 (12)	0.0318 (4)	
C10	0.2425 (3)	0.1306 (2)	0.33572 (13)	0.0420 (4)	
H10A	0.2271	0.2098	0.2993	0.050*	
C11	0.2835 (3)	0.0109 (2)	0.28654 (14)	0.0460 (5)	
H11A	0.2955	0.0092	0.2158	0.055*	
C12	0.3070 (3)	−0.1066 (2)	0.34058 (14)	0.0438 (5)	
H12A	0.3348	−0.1860	0.3053	0.053*	
C13	0.2903 (3)	−0.10923 (18)	0.44609 (13)	0.0346 (4)	
H13A	0.3066	−0.1887	0.4822	0.042*	
C14	0.2484 (2)	0.01083 (17)	0.49553 (12)	0.0281 (3)	
C15	0.1837 (2)	0.15576 (16)	0.62770 (12)	0.0286 (3)	

### Atomic displacement parameters (Å^2^)

**Table:**

	*U*^11^	*U*^22^	*U*^33^	*U*^12^	*U*^13^	*U*^23^
Mn1	0.0344 (2)	0.01988 (18)	0.02706 (18)	0.00148 (14)	0.00271 (14)	−0.00338 (13)
S1	0.0469 (3)	0.0276 (2)	0.0324 (2)	−0.00140 (19)	−0.00021 (18)	0.00664 (17)
N1	0.0333 (7)	0.0255 (7)	0.0245 (6)	−0.0037 (6)	−0.0022 (5)	0.0037 (5)
N2	0.0529 (9)	0.0294 (8)	0.0276 (7)	−0.0034 (7)	0.0011 (6)	−0.0010 (6)
O1	0.0524 (8)	0.0284 (6)	0.0259 (6)	−0.0013 (5)	0.0028 (5)	0.0018 (5)
O1W	0.0639 (10)	0.0472 (9)	0.0544 (9)	−0.0090 (8)	0.0123 (7)	−0.0130 (7)
O2	0.0485 (7)	0.0245 (6)	0.0308 (6)	0.0045 (5)	0.0086 (5)	0.0026 (5)
O2W	0.0571 (9)	0.0365 (8)	0.0530 (9)	−0.0023 (7)	0.0064 (7)	−0.0124 (6)
O3	0.0376 (7)	0.0359 (7)	0.0449 (7)	−0.0064 (5)	−0.0048 (5)	−0.0132 (6)
O3W	0.0619 (11)	0.0738 (12)	0.0557 (10)	0.0070 (9)	−0.0036 (8)	−0.0071 (9)
O4	0.0322 (6)	0.0270 (6)	0.0461 (7)	0.0012 (5)	−0.0041 (5)	−0.0074 (5)
O5	0.0380 (6)	0.0235 (6)	0.0276 (5)	0.0021 (5)	−0.0013 (5)	−0.0050 (4)
C1	0.0276 (8)	0.0256 (8)	0.0274 (7)	−0.0030 (6)	0.0052 (6)	0.0024 (6)
C2	0.0273 (8)	0.0218 (7)	0.0272 (7)	0.0012 (6)	0.0043 (6)	0.0002 (6)
C3	0.0314 (8)	0.0238 (8)	0.0344 (8)	−0.0045 (6)	−0.0023 (6)	0.0000 (6)
C4	0.0362 (9)	0.0313 (9)	0.0381 (9)	−0.0001 (7)	−0.0094 (7)	0.0004 (7)
C5	0.0460 (10)	0.0268 (8)	0.0297 (8)	−0.0014 (7)	−0.0050 (7)	0.0046 (6)
C6	0.0339 (8)	0.0249 (8)	0.0255 (7)	−0.0025 (6)	0.0029 (6)	0.0015 (6)
C7	0.0307 (8)	0.0178 (7)	0.0272 (7)	−0.0023 (6)	0.0006 (6)	−0.0010 (6)
C8	0.0324 (8)	0.0261 (8)	0.0256 (7)	−0.0060 (7)	0.0017 (6)	−0.0017 (6)
C9	0.0303 (8)	0.0358 (9)	0.0289 (8)	−0.0038 (7)	−0.0031 (6)	0.0027 (7)
C10	0.0423 (10)	0.0552 (12)	0.0284 (8)	−0.0067 (9)	−0.0033 (7)	0.0094 (8)
C11	0.0425 (11)	0.0701 (14)	0.0257 (8)	−0.0088 (10)	−0.0021 (7)	−0.0047 (9)
C12	0.0360 (10)	0.0560 (12)	0.0399 (10)	−0.0073 (9)	0.0010 (8)	−0.0181 (9)
C13	0.0310 (9)	0.0358 (9)	0.0373 (9)	−0.0051 (7)	−0.0026 (7)	−0.0057 (7)
C14	0.0247 (8)	0.0334 (9)	0.0263 (7)	−0.0045 (6)	−0.0021 (6)	0.0002 (6)
C15	0.0293 (8)	0.0273 (8)	0.0292 (8)	−0.0037 (6)	−0.0024 (6)	0.0027 (6)

### Geometric parameters (Å, °)

**Table:**

Mn1—O4	2.1083 (11)	C1—C2	1.522 (2)
Mn1—O4^i^	2.1083 (11)	C2—C3	1.532 (2)
Mn1—O2	2.1883 (11)	C2—C7	1.581 (2)
Mn1—O2^i^	2.1883 (11)	C2—H2A	0.9800
Mn1—O5^i^	2.2598 (11)	C3—C4	1.523 (2)
Mn1—O5	2.2598 (11)	C3—H3A	0.9800
S1—C15	1.7354 (16)	C4—C5	1.542 (3)
S1—C9	1.7519 (18)	C4—H4A	0.9700
N1—C15	1.323 (2)	C4—H4B	0.9700
N1—C14	1.392 (2)	C5—C6	1.521 (2)
N1—H1N	0.841 (15)	C5—H5A	0.9700
N2—C15	1.313 (2)	C5—H5B	0.9700
N2—H2B	0.8600	C6—C7	1.538 (2)
N2—H2C	0.8600	C6—H6A	0.9800
O1—C1	1.2465 (19)	C7—C8	1.532 (2)
O1W—H1WA	0.812 (16)	C7—H7A	0.9800
O1W—H1WB	0.847 (16)	C9—C10	1.388 (2)
O2—C1	1.2729 (19)	C9—C14	1.391 (2)
O2W—H2WA	0.826 (16)	C10—C11	1.377 (3)
O2W—H2WB	0.835 (16)	C10—H10A	0.9300
O3—C8	1.2397 (19)	C11—C12	1.381 (3)
O3W—H3WA	0.802 (17)	C11—H11A	0.9300
O3W—H3WB	0.823 (17)	C12—C13	1.387 (3)
O4—C8	1.268 (2)	C12—H12A	0.9300
O5—C6	1.4577 (19)	C13—C14	1.382 (2)
O5—C3	1.462 (2)	C13—H13A	0.9300
			
O4—Mn1—O4^i^	180.00 (7)	C5—C4—H4A	111.4
O4—Mn1—O2	86.35 (5)	C3—C4—H4B	111.4
O4^i^—Mn1—O2	93.65 (5)	C5—C4—H4B	111.4
O4—Mn1—O2^i^	93.65 (5)	H4A—C4—H4B	109.3
O4^i^—Mn1—O2^i^	86.35 (5)	C6—C5—C4	101.69 (13)
O2—Mn1—O2^i^	180.00 (8)	C6—C5—H5A	111.4
O4—Mn1—O5^i^	95.79 (4)	C4—C5—H5A	111.4
O4^i^—Mn1—O5^i^	84.21 (4)	C6—C5—H5B	111.4
O2—Mn1—O5^i^	94.16 (4)	C4—C5—H5B	111.4
O2^i^—Mn1—O5^i^	85.84 (4)	H5A—C5—H5B	109.3
O4—Mn1—O5	84.21 (4)	O5—C6—C5	102.21 (13)
O4^i^—Mn1—O5	95.79 (4)	O5—C6—C7	102.25 (11)
O2—Mn1—O5	85.84 (4)	C5—C6—C7	111.05 (13)
O2^i^—Mn1—O5	94.16 (4)	O5—C6—H6A	113.4
O5^i^—Mn1—O5	180.00 (9)	C5—C6—H6A	113.4
C15—S1—C9	90.04 (8)	C7—C6—H6A	113.4
C15—N1—C14	114.40 (13)	C8—C7—C6	110.03 (13)
C15—N1—H1N	120.2 (15)	C8—C7—C2	115.94 (12)
C14—N1—H1N	125.4 (15)	C6—C7—C2	100.67 (12)
C15—N2—H2B	120.0	C8—C7—H7A	109.9
C15—N2—H2C	120.0	C6—C7—H7A	109.9
H2B—N2—H2C	120.0	C2—C7—H7A	109.9
H1WA—O1W—H1WB	109 (2)	O3—C8—O4	123.92 (16)
C1—O2—Mn1	117.36 (10)	O3—C8—C7	118.49 (14)
H2WA—O2W—H2WB	110 (2)	O4—C8—C7	117.49 (13)
H3WA—O3W—H3WB	112 (2)	C10—C9—C14	120.86 (17)
C8—O4—Mn1	130.31 (11)	C10—C9—S1	128.60 (15)
C6—O5—C3	95.66 (11)	C14—C9—S1	110.53 (12)
C6—O5—Mn1	117.76 (9)	C11—C10—C9	117.93 (18)
C3—O5—Mn1	112.38 (9)	C11—C10—H10A	121.0
O1—C1—O2	123.97 (15)	C9—C10—H10A	121.0
O1—C1—C2	117.61 (14)	C10—C11—C12	121.05 (17)
O2—C1—C2	118.42 (13)	C10—C11—H11A	119.5
C1—C2—C3	113.69 (13)	C12—C11—H11A	119.5
C1—C2—C7	113.13 (13)	C11—C12—C13	121.61 (18)
C3—C2—C7	101.24 (12)	C11—C12—H12A	119.2
C1—C2—H2A	109.5	C13—C12—H12A	119.2
C3—C2—H2A	109.5	C14—C13—C12	117.40 (17)
C7—C2—H2A	109.5	C14—C13—H13A	121.3
O5—C3—C4	101.85 (13)	C12—C13—H13A	121.3
O5—C3—C2	102.11 (12)	C13—C14—C9	121.14 (15)
C4—C3—C2	111.14 (13)	C13—C14—N1	126.60 (15)
O5—C3—H3A	113.5	C9—C14—N1	112.26 (14)
C4—C3—H3A	113.5	N2—C15—N1	124.04 (15)
C2—C3—H3A	113.5	N2—C15—S1	123.19 (13)
C3—C4—C5	101.90 (14)	N1—C15—S1	112.76 (12)
C3—C4—H4A	111.4		
			
O4—Mn1—O2—C1	36.93 (12)	Mn1—O5—C6—C7	−60.82 (13)
O4^i^—Mn1—O2—C1	−143.07 (12)	C4—C5—C6—O5	34.93 (15)
O5^i^—Mn1—O2—C1	132.48 (12)	C4—C5—C6—C7	−73.49 (16)
O5—Mn1—O2—C1	−47.52 (12)	O5—C6—C7—C8	87.29 (14)
O2—Mn1—O4—C8	−55.66 (14)	C5—C6—C7—C8	−164.32 (13)
O2^i^—Mn1—O4—C8	124.34 (14)	O5—C6—C7—C2	−35.56 (14)
O5^i^—Mn1—O4—C8	−149.48 (14)	C5—C6—C7—C2	72.82 (15)
O5—Mn1—O4—C8	30.52 (14)	C1—C2—C7—C8	3.37 (18)
O4—Mn1—O5—C6	12.07 (10)	C3—C2—C7—C8	−118.65 (14)
O4^i^—Mn1—O5—C6	−167.93 (10)	C1—C2—C7—C6	122.02 (14)
O2—Mn1—O5—C6	98.81 (10)	C3—C2—C7—C6	−0.01 (14)
O2^i^—Mn1—O5—C6	−81.19 (10)	Mn1—O4—C8—O3	168.08 (12)
O4—Mn1—O5—C3	−97.64 (10)	Mn1—O4—C8—C7	−15.5 (2)
O4^i^—Mn1—O5—C3	82.36 (10)	C6—C7—C8—O3	127.93 (15)
O2—Mn1—O5—C3	−10.90 (9)	C2—C7—C8—O3	−118.70 (16)
O2^i^—Mn1—O5—C3	169.10 (9)	C6—C7—C8—O4	−48.67 (18)
Mn1—O2—C1—O1	−134.48 (14)	C2—C7—C8—O4	64.70 (18)
Mn1—O2—C1—C2	45.82 (17)	C15—S1—C9—C10	179.41 (17)
O1—C1—C2—C3	−156.51 (14)	C15—S1—C9—C14	−0.48 (13)
O2—C1—C2—C3	23.2 (2)	C14—C9—C10—C11	0.0 (3)
O1—C1—C2—C7	88.73 (17)	S1—C9—C10—C11	−179.86 (14)
O2—C1—C2—C7	−91.56 (17)	C9—C10—C11—C12	−0.2 (3)
C6—O5—C3—C4	56.86 (13)	C10—C11—C12—C13	0.1 (3)
Mn1—O5—C3—C4	−179.98 (9)	C11—C12—C13—C14	0.2 (3)
C6—O5—C3—C2	−58.10 (13)	C12—C13—C14—C9	−0.3 (2)
Mn1—O5—C3—C2	65.06 (12)	C12—C13—C14—N1	179.42 (16)
C1—C2—C3—O5	−86.20 (14)	C10—C9—C14—C13	0.2 (3)
C7—C2—C3—O5	35.44 (14)	S1—C9—C14—C13	−179.86 (13)
C1—C2—C3—C4	165.85 (13)	C10—C9—C14—N1	−179.55 (16)
C7—C2—C3—C4	−72.52 (16)	S1—C9—C14—N1	0.36 (17)
O5—C3—C4—C5	−35.17 (15)	C15—N1—C14—C13	−179.74 (16)
C2—C3—C4—C5	72.95 (17)	C15—N1—C14—C9	0.0 (2)
C3—C4—C5—C6	0.24 (16)	C14—N1—C15—N2	−179.49 (15)
C3—O5—C6—C5	−56.87 (13)	C14—N1—C15—S1	−0.41 (18)
Mn1—O5—C6—C5	−175.85 (9)	C9—S1—C15—N2	179.61 (15)
C3—O5—C6—C7	58.16 (13)	C9—S1—C15—N1	0.51 (13)

Symmetry codes: (i) −*x*+2, −*y*, −*z*.

### Hydrogen-bond geometry (Å, °)

**Table:**

*D*—H···*A*	*D*—H	H···*A*	*D*···*A*	*D*—H···*A*
N1—H1N···O1^ii^	0.84 (2)	1.84 (2)	2.6822 (18)	178 (2)
N2—H2B···O2^ii^	0.86	2.00	2.8490 (19)	170
N2—H2C···O2W^iii^	0.86	2.00	2.824 (2)	160
O1W—H1WA···O1	0.81 (2)	2.03 (2)	2.8250 (19)	166 (3)
O1W—H1WB···O2W	0.85 (2)	1.95 (2)	2.792 (2)	170 (3)
O2W—H2WA···O3	0.83 (2)	1.87 (2)	2.6806 (19)	169 (3)
O2W—H2WB···O3W^iv^	0.84 (2)	1.93 (2)	2.768 (2)	178 (3)
O3W—H3WA···O1W^iii^	0.80 (2)	2.21 (2)	3.004 (2)	169 (3)
O3W—H3WB···O1W	0.82 (2)	1.97 (2)	2.784 (2)	173 (3)

Symmetry codes: (ii) −*x*+1, −*y*, −*z*+1; (iii) −*x*+1, −*y*+1, −*z*+1; (iv) *x*+1, *y*, *z*.

## References

[bb1] Bruker (2006). *APEX2* and *SAINT* Bruker AXS Inc., Madison, Wisconsin, USA.

[bb2] Dukhande, V. V., Malthankar-Phatak, G. H., Hugus, J. J., Daniels, C. K. & Lai, J. C. K. (2006). *Neurochem. Res.* **31**, 1349–1357. 10.1007/s11064-006-9179-7 17053969

[bb3] Sheldrick, G. M. (1996). *SADABS* University of Göttingen, Germany.

[bb4] Sheldrick, G. M. (2008). *Acta Cryst.* A**64**, 112–122. 10.1107/S0108767307043930 18156677

[bb5] Shimi, I. R., Zaki, Z., Shoukry, S. & Medhat, A. M. (1982). *Eur. J. Cancer Clin. Oncol.* **18**, 785–789. 10.1016/0277-5379(82)90078-5 6891327

[bb6] Wang, N., Lin, Q.-Y., Feng, J., Li, S.-K. & Zhao, J.-J. (2010). *Acta Cryst.* E**66**, m763–m764. 10.1107/S1600536810020921 PMC3006894 21587697

